# Immune Subtyping Identifies Patients With Hormone Receptor–Positive Early-Stage Breast Cancer Who Respond to Neoadjuvant Immunotherapy (IO): Results From Five IO Arms of the I-SPY2 Trial

**DOI:** 10.1200/PO-24-00776

**Published:** 2025-06-17

**Authors:** Denise M. Wolf, Christina Yau, Michael Campbell, Annuska Glas, Andrei Barcaru, Lorenza Mittempergher, Midas Kuilman, Lamorna Brown-Swigart, Gillian Hirst, Amrita Basu, Mark Magbanua, Rosalyn Sayaman, Laura Huppert, Amy Delson, I-SPY2 Investigators, W. Fraser Symmans, Alexander Borowsky, Paula Pohlmann, Hope Rugo, Amy Clark, Douglas Yee, Angela DeMichele, Jane Perlmutter, Emmanuel F. Petricoin, Jo Chien, Erica Stringer-Reasor, Rebecca Shatsky, Minetta Liu, Hyo Han, Hatem Soliman, Claudine Isaacs, Rita Nanda, Nola Hylton, Lajos Pusztai, Laura Esserman, Laura van ‘t Veer

**Affiliations:** ^1^ Department of Laboratory Medicine, University of California San Francisco, San Francisco, CA; ^2^ Department of Surgery, University of California San Francisco, San Francisco, CA; ^3^ Agendia Inc, Irvine, CA; ^4^ Division of Hematology/Oncology, University of California San Francisco, San Francisco, CA; ^5^ Breast Science Advocacy Core, University of California San Francisco, San Francisco, CA; ^6^ Department of Pathology, University of Texas MD Anderson Cancer Center, Houston, TX; ^7^ Department of Pathology, University of California Davis, Davis, CA; ^8^ Division of Cancer Medicine, University of Texas MD Anderson Cancer Center, Houston, TX; ^9^ Perelman School of Medicine, University of Pennsylvania, Philadelphia, PA; ^10^ Department of Medicine, University of Minnesota, Minneapolis, MN; ^11^ Gemini Group, Ann Arbor, MI; ^12^ Center for Applied Proteomics, George Mason University, Manassas, VA; ^13^ Department of Medicine, University of Alabama, Birmingham, AL; ^14^ Department of Medicine, University of California San Diego, San Diego, CA; ^15^ Department of Oncology, Mayo Clinic, Rochester, MN; ^16^ Department of Medical Oncology, Moffitt Cancer Center, Tampa, FL; ^17^ Department of Medicine, Georgetown University, Washington, DC; ^18^ Department of Medicine, University of Chicago, Chicago, IL; ^19^ Department of Radiology, University of California San Francisco, San Francisco, CA; ^20^ Yale School of Medicine, Yale University, New Haven, CT

## Abstract

**PURPOSE:**

Neoadjuvant immunotherapy (IO) has become the standard of care for early-stage triple-negative breast cancer (TNBC), but not yet for other subtypes. We previously developed a clinical-grade mRNA-based immune classifier (ImPrint) predicting response to IO that is now being used in I-SPY2.2 as part of the response predictive subtypes. We report the performance of ImPrint in hormone receptor–positive and human epidermal growth factor receptor 2–negative (HR+HER2–) patients from five IO arms.

**METHODS:**

A total of 204 HR+HER2– (MammaPrint high-risk) patients from five IO arms (anti–PD-1, anti–PD-L1/poly [ADP-ribose] polymerase inhibitor combination, anti–PD-1/toll-like receptor 9 dual-IO combination, and anti–PD-1 ± lymphocyte activation gene 3 dual-IO combination) and 191 patients from the chemotherapy-only control arm were included in this analysis. Patients were classified as ImPrint+ (likely sensitive) versus ImPrint– (likely resistant), using pretreatment mRNA. Performance of ImPrint for predicting pathologic complete response (pCR) to IO-containing arms was characterized and compared with tumor grade (III), MammaPrint (ultra) High2 risk (MP2), and estrogen receptor (ER)-low (ER ≤ 10%).

**RESULTS:**

Overall, the pCR rate across the five IO arms was 33%. 26% of HR+HER2– patients were ImPrint+, and pCR rates with IO were 75% in ImPrint+ versus 17% in ImPrint–, with the highest pCR rate >90% in a dual-IO arm. In the control arm, pCR rates were 33% in ImPrint+ and 8% in ImPrint–. Tumor grade (III), MP2, and ER-low showed pCR rates in IO of 45%, 56%, and 63%, respectively, with lower pCR odds ratios (OR < 7.5) compared with ImPrint (OR = 14.5).

**CONCLUSION:**

Using an accurate selection strategy, HR+HER2– patients could achieve pCR rates similar to what is seen with best neoadjuvant therapies in TNBC and HER2+ (ie, pCR rate >65%-70%). ImPrint, an Food and Drug Administration IDE-enabled assay, may represent a way to identify HR+HER2– patients for IO that best balances likely benefit versus risk of serious immune-related adverse events.

## INTRODUCTION

Neoadjuvant immunotherapy (IO) has become the standard of care for patients with early-stage triple-negative breast cancer (TNBC), but not yet for other breast cancer subtypes. Most initial IO clinical trials excluded patients with early-stage hormone receptor–positive and human epidermal growth factor receptor 2–negative (HR+HER2–) breast cancers, considered unlikely to respond because of low levels of immune infiltrate. However, some HR+HER2– cancers have high levels of immune infiltration, like TNBC,^1^ and accumulating evidence suggests there may be a subset of patients with HR+/HER2– disease that derive benefit from IO. The I-SPY2 trial (ClinicalTrials.gov identifier: NCT01042379) was the first randomized trial to examine the efficacy of IO therapy in patients with high-risk HR+HER2– breast cancers, followed by KEYNOTE-756^2^ and CheckMate-7FL.^3^

**CONTEXT**

**Key Objective**
I-SPY2 was the first randomized trial to show that neoadjuvant immunotherapy (IO) improved efficacy relative to a chemotherapy-only arm in high-risk early-stage hormone receptor–positive and human epidermal growth factor receptor 2–negative (HR+HER2–) breast cancer. Given that the response rates were relatively low and serious immune-related toxicities are a risk, a predictive biomarker is needed to identify HR+HER2– patients who are highly sensitive to IO.
**Knowledge Generated**
We show that ImPrint, an Food and Drug Administration IDE-enabled gene expression–based immune classifier developed in the first anti–PD-1 IO arm in I-SPY2, validates as highly predictive of response for HR+HER2– to IO in four subsequent independent IO arms.
**Relevance**
ImPrint may be a good biomarker to help allocate IO therapy to HR+HER2– patients most likely to benefit, as the benefits of IO for ImPrint+ patients (75% pathologic complete response [pCR]) seem to greatly outweigh the risks of serious toxicities. For ImPrint– patients, the low benefit of adding IO (17% pCR) likely does not outweigh potential toxicities.

**FIG 1.**
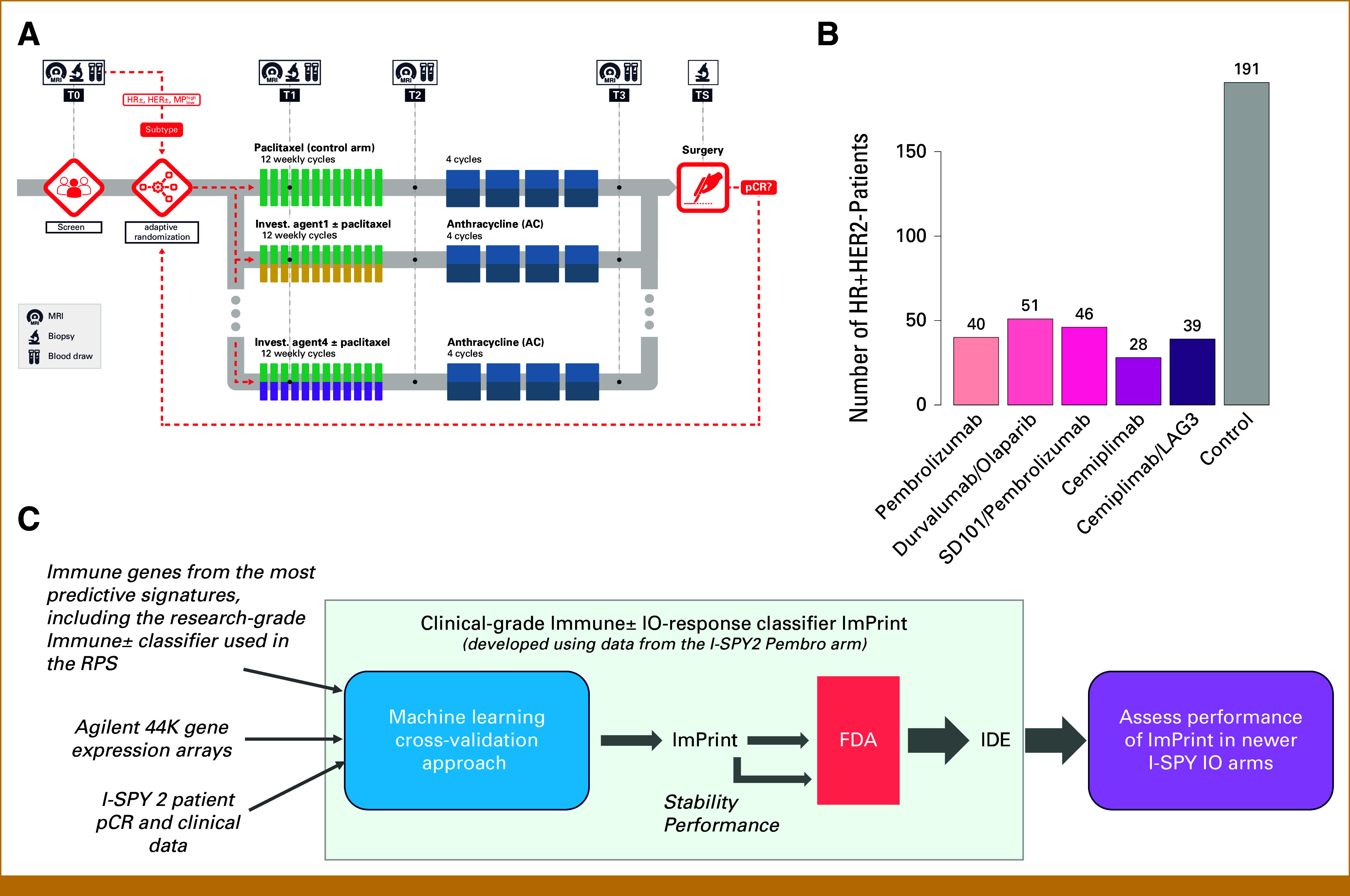
Trial design and data. (A) I-SPY2 trial schematic, (B) patients per arm in this analysis, (C) development of the IO response prediction clinical biomarker ImPrint. AC, doxorubicin/cyclophosphamide; FDA, Food and Drug Administration; HER2, human epidermal growth factor receptor 2; HR, hormone receptor; IDE, investigational device exemption; IO, immunotherapy; LAG3, lymphocyte activation gene 3; MP, MammaPrint; pCR, pathologic complete response; RPS, response predictive subtypes.

The I-SPY2 trial is an ongoing multicenter, phase II neoadjuvant platform trial for patients with high-risk, early-stage breast cancer designed to rapidly identify new treatments and treatment combinations with increased efficacy compared with the standard of care (sequential weekly paclitaxel [T] followed by doxorubicin/cyclophosphamide [AC] chemotherapy; Fig 1A). In I-SPY2, multiple investigational treatment regimens are simultaneously and adaptively randomized against the shared control arm.^4-10^ The primary efficacy end point is pathologic complete response (pCR), defined as no invasive cancer in the breast and lymph nodes at the time of surgery.^11,12^ Patients with pCR have excellent long-term recurrence-free survival in all breast cancer subtypes, including HR+/HER2– cancers.^12^ Among patients with HR+HER2– early-stage breast cancer, only those with MammaPrint (MP) high-risk disease are eligible for the trial.

In I-SPY2, HR+HER2– patients are eligible for all IO arms. In this study, we focus on IO response predictive biomarkers in patients with HR+HER2– disease from five IO arms, all given concurrently with paclitaxel and followed by AC chemotherapy without IO (Fig 1B). These include two arms with the anti–PD-1 agents pembrolizumab^7^ or cemiplimab^13^; one arm with an anti–PD-L1 antibody, durvalumab, combined with the poly (ADP-ribose) polymerase (PARP) inhibitor olaparib^10^; and two dual-IO arms, one combining pembrolizumab with a toll-like receptor 9 agonist^14^ and the other combining cemiplimab with a lymphocyte activation gene 3 (LAG3) inhibitor.^15^ Four of the five arms graduated for efficacy in HR+HER2– patients. However, most patients with HR+HER2– disease did not achieve a pCR and IO agents carry substantial risk of long-term immune-related toxicities and represent a major health care cost.^16^ This has motivated us to develop more accurate predictors of IO benefit in HR+HER2– breast cancer.

We previously identified a research-grade dichotomous mRNA expression-based biomarker capturing tumor-immune biology associated with response to IO, which we incorporated into our novel breast cancer response-predictive subtyping system.^17^ We translated the candidate marker set into a clinical-grade immune classifier, ImPrint, that has received investigational device exeption (IDE) approval from the US Food and Drug Administration (FDA) and is now being prospectively tested in I-SPY2 (Fig 1C). We report the performance of ImPrint to predict pCR to neoadjuvant IO therapy in HR+HER2– patients, including the IO discovery arm and four independent IO validation arms of I-SPY2.

## METHODS

### I-SPY2 Trial Overview

I-SPY2 is an ongoing, open-label, adaptive, randomized phase II multicenter clinical trial of neoadjuvant therapy for patients with early-stage breast cancer (ClinicalTrials.gov identifier: NCT01042379). Details on the study design and previous biomarker work have been published earlier.^4-10,17-24^

### Investigational Regimens Included in This Analysis

Since the I-SPY2 trial began in 2010, 23 investigational regimens have been evaluated to date. This analysis includes data from patients with HR+HER2– disease (clinically positive for estrogen receptor [ER] or/and progesterone receptor [PGR]; and HER2-negative) who were enrolled in the paclitaxel control arm before December 9, 2021, and in five previously reported IO arms with paclitaxel plus an investigational PD-1/PD-L1 agent or combination: (1) pembrolizumab,^7^ (2) durvalumab + olaparib,^10^ (3) pembrolizumab +SD101,^14^ and (4-5) cemiplimab ± LAG3.^13,15^ All patients subsequently received an anthracycline regimen per protocol.

### Trial Oversight

I-SPY2 is conducted in accordance with the guidelines for Good Clinical Practice and the Declaration of Helsinki, with approval for the study protocol and associated amendments obtained from independent ethics committees at each site (pembrolizumab, durvalumab + olaparib and pembrolizumab + SD101 arms) or by the Wake Forest Central Institutional Review Board for all arms enrolling after 2020 (cemiplimab ± LAG3 arms). Written informed consent was obtained from each participant before screening and again before treatment. The I-SPY2 Data Safety Monitoring Board meets monthly to review patient safety.

### Pretreatment Biopsy Processing and Molecular Profiling

Sixteen-gauge core needle biopsies were obtained from the primary breast tumor before treatment. Tissue sections (fresh frozen [FF] or formalin-fixed paraffin-embedded [FFPE]) were processed at Agendia Inc, Irvine, CA, for RNA extraction and profiling on Agilent 32K (Agendia32627_DPv1.14_SCFGplus, annotation GPL20078) gene expression arrays under IDE in their Clinical Laboratory Improvement Amendments (CLIA)–certified clinical laboratory, as previously described.^17^

### ImPrint-Positive Versus ImPrint-Negative Classification

Patients were classified using ImPrint, a 53-gene expression–based immune-response predictive biomarker, as ImPrint positive (likely sensitive) versus ImPrint negative (likely resistant), by Agendia, Inc, blinded to outcome data. This classifier was originally developed using data from the first pembrolizumab + paclitaxel arm of I-SPY2 using a cross-validation machine learning algorithm applied to a list of immune genes identified from previous correlative analysis (Fig 1C).^25,26^

### Statistical Methods

Performance of ImPrint for predicting pCR to IO was characterized by calculating (1) pCR rates in ImPrint+ versus ImPrint– groups; (2) odds ratios (ORs) for achieving pCR in ImPrint+ versus ImPrint– and associated *P* values using Fisher's exact test; and (3) sensitivity, specificity, positive predictive value (PPV), and negative predictive value (NPV). To assess relative performance in IO versus the control arm, we also calculated the above (1-3) for the control arm, as well as the *P* value for the biomarker × treatment-class interaction. Performance of MP (ultra) High2 risk (MP2), grade III, and ER-low (ER ≤ 10%) was assessed as well and compared with ImPrint.

## RESULTS

### Demographic and Tumor Characteristics

This analysis includes 395 patients with HR+HER2– early-stage breast cancer who participated in I-SPY2 (n = 191 in control and n = 204 from five IO arms: pembrolizumab [n = 40], durvalumab/olaparib [n = 51], pembrolizumab/SD101 [n = 46], cemiplimab [n = 28], and cemiplimab/LAG3 [n = 39]; Fig 1B). Demographic and tumor characteristics are listed in Table 1. When we compared the combined five IO arms with the control arm, there was no significant difference in patient age, race/ethnicity, menopausal status, grade, tumor longest diameter (LD) by magnetic resonance image (MR), tumor stage, ER-low, or nodal stage (Table 1; right-most column). However, there were significantly more patients classified as MP2 in the IO arms (91/204; 45%) compared with that in the control arm (59/191; 31%), likely reflecting the action of the I-SPY2 adaptive randomization engine over HR/HER2/MP-defined subtypes (preferentially randomizing in favor of MP2 status in IO).

**TABLE 1.** Patient Characteristics

Clinical/Demographic Variable	Overall (n = 395), N (%) Unless Otherwise Indicated	Five IO Arms (n = 204), N (%) Unless Otherwise Indicated	Control Arm (n = 191), N (%) Unless Otherwise Indicated	*P* (Five IO Arms *v* Control; Fisher's Exact Test or *t* Test)
Age, years				
Median (range)	47 (19-71)	47 (21-71)	48 (19-71)	.52
Race/ethnicity				
White (non-Hispanic)	270 (68)	135 (66)	135 (71)	.53
White (Hispanic)	51 (13)	30 (15)	21 (11)	
Black	43 (11)	20 (10)	23 (12)	
Asian	25 (6)	14 (7)	11 (6)	
American Indian or Alaska Native	2 (0.5)	2 (1)	0 (0)	
Multiple races	2 (0.5)	1 (0.5)	1 (0.5)	
Unknown	2 (0.5)	2 (1)	0 (0)	
Menopausal status				
Premenopausal/perimenopausal (or age <50)	238 (60)	125 (39)	113 (59)	.68
Postmenopausal (or age >50)	157 (40)	79 (39)	78 (41)	
Tumor longest diameter on MR, cm				
Median (range)	3.9 (1.3-13)	4.1 (1.3-13)	3.8 (1.4-12)	.67
Tumor stage (T)				
T1	12 (3)	3 (1.5)	9 (5)	.31
T2	238 (62)	127 (63)	111 (60)	
T3	126 (33)	66 (33)	60 (32)	
T4	10 (2.6)	5 (2.5)	5 (3)	
Unknown	9	3	6	
Nodal stage (N)				
N0	135 (35)	66 (33)	69 (38)	.74
N1	204 (53)	110 (55)	94 (51)	
N2	20 (5)	12 (6)	8 (4)	
N3	25 (7)	13 (7)	12 (7)	
Unknown	11	3	8	
MP status				
MP1	245 (62)	113 (55)	132 (69)	**.0052**
MP2	150 (38)	91 (45)	59 (31)	
Tumor grade (available for n = 282/395)				
Grade I	5 (2)	3 (2)	2 (1.5)	.40 (*P* for known *v* unknown grade = .27)
Grade II	108 (38)	53 (35)	55 (42)	
Grade III	169 (60)	95 (63)	74 (56.5)	
Unknown	113	53	60	
ImPrint status				
ImPrint–	291 (74)	145 (71)	146 (76)	.25
ImPrint+	104 (26)	59 (29)	45 (24)	
% ER+ cells (available for n = 386/395)				
ER ≤ 10%	58 (15)	35 (17)	23 (12)	.15
ER > 10%	328 (85)	162 (83)	166 (88)	
Unknown	9	7	2	

NOTE. Bold indicates statistical significant values.

Abbreviations: ER, estrogen receptor; IO, immunotherapy; MP1, MammaPrint High 1 risk; MP2, MammaPrint (ultra) High2 risk; MR, magnetic resonance (image).

### Prevalence of ImPrint in HR+HER2– and Association With Demographic and Tumor Characteristics

**FIG 2.**
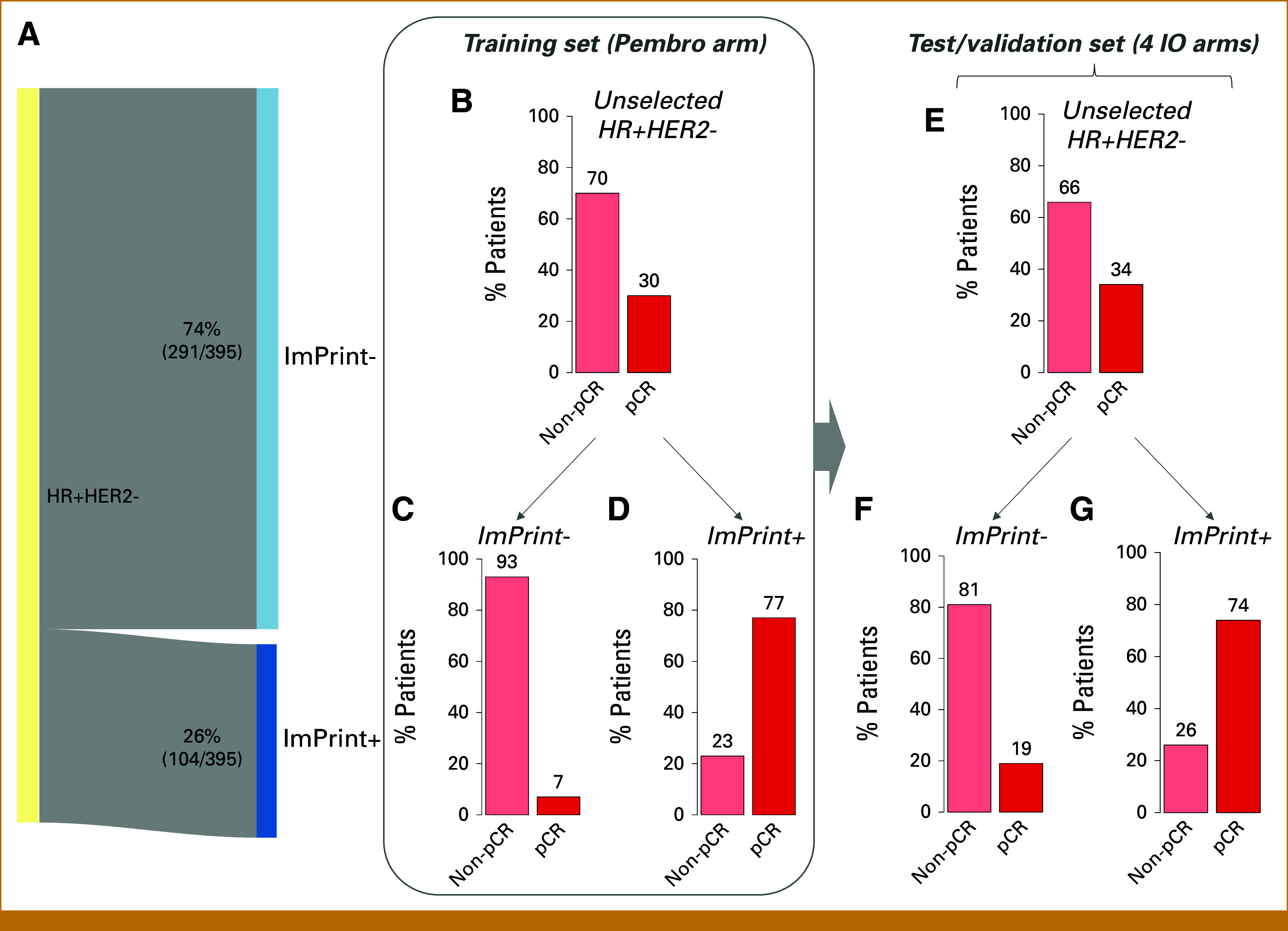
Prevalence and performance of ImPrint for HR+HER2–. (A) Sankey diagram showing prevalence of ImPrint+ versus ImPrint– in HR+HER2– in I-SPY2. (B-D) pCR bar plots in unselected HR+HER2– (B) versus in ImPrint– (C) and ImPrint+ (D) in the IO arm used as a training set (pembro). (E -G) pCR bar plots in unselected HR+HER2– (E) versus in ImPrint– (F) and ImPrint+ (G) in the four IO arms used as independent validation sets (durvalumab/olaparib, pembrolizumab/SD101, cemiplimab, and cemiplimab/LAG3). HR+HER2–, hormone receptor–positive and human epidermal growth factor receptor 2–negative; IO, immunotherapy; LAG3, lymphocyte activation gene 3; pCR, pathologic complete response.

**FIG A1.**
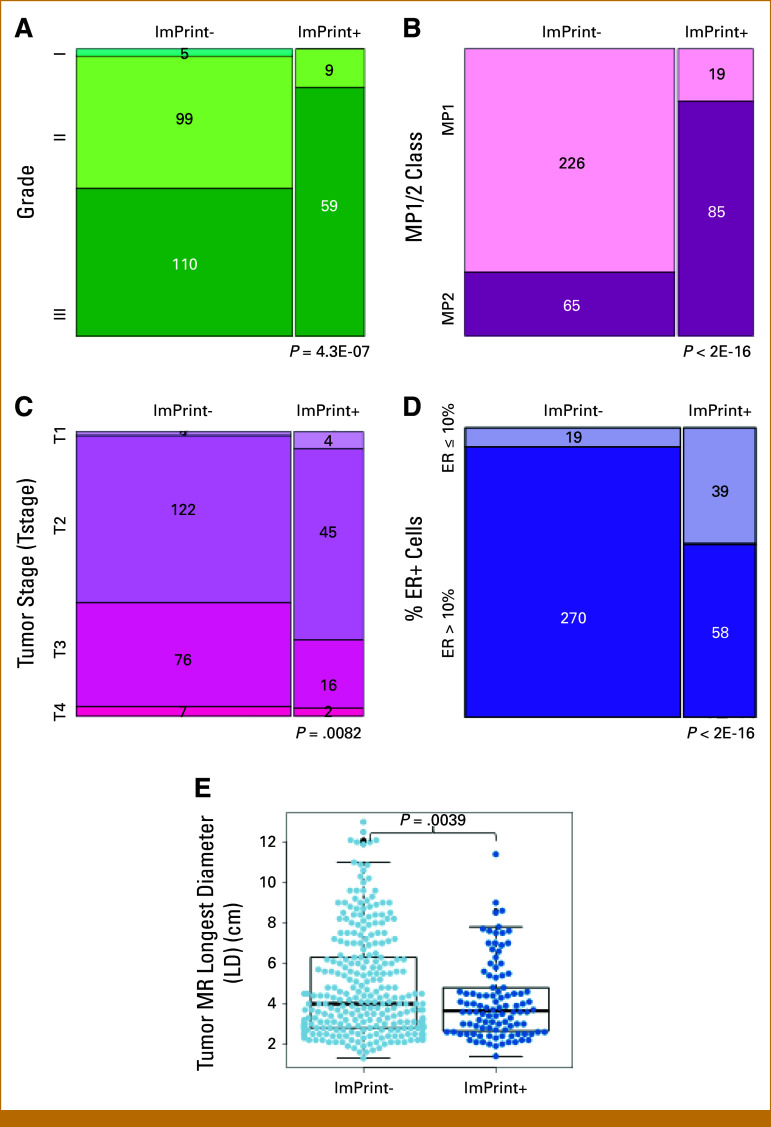
Tumor clinical and molecular features associated with ImPrint status in the HR+HER2– subset in I-SPY2. (A-D) Mosaic plots showing the distribution of tumor grade (A), MP1/2 class (B), tumor stage (C), and % ER+ cells (D) in ImPrint– versus ImPrint+ subsets (*P* values from Fisher's exact tests). (E) Box plot showing tumor LD by MR in ImPrint– versus ImPrint+ subsets (*P* value from a *t* test). Boxes show median and 25th-75th IQR. Whiskers denote largest/smallest values within 1.5× the IQR. ER, estrogen receptor; HR+HER2–, hormone receptor–positive and human epidermal growth factor receptor 2–negative; LD, longest diameter; MP1, MammaPrint High 1 risk; MP2, MammaPrint (ultra) High2 risk; MR, magnetic resonance (imaging).

In this cohort, 26% (104/395) of HR+HER2– patients were classified as ImPrint+ and thus predicted to respond to IO (Fig 2A). ImPrint+ prevalence did not significantly differ between the IO arms and control arm (Table 1). Although we hypothesized that patients with tumors classified as ImPrint+ might be younger and more commonly premenopausal/perimenopausal than those with ImPrint– tumors, because of previously reported association between higher immune infiltration and premenopausal status in women with low genomic risk, ER+HER2– cancers,^27^ we observed no significant difference in age, menopausal status, or race/ethnicity between ImPrint± classes (Appendix Table A1). Consistent with prior observations that high levels of intratumoral immune infiltrate are associated with TNBC/basal biology and high proliferation,^10,28,29^ ImPrint+ tumors had significantly higher grade (87% grade III), MP2 class (82% MP2), and ER-low (40% ER-low) prevalence than ImPrint– tumors (51% grade III; 22% MP2; 7% ER-low) (Appendix Figs A1A, A1B, A1D and Table A1). Interestingly, ImPrint+ tumors were significantly smaller (77% with T-stage = T1/T2) than ImPrint– tumors (60% with T-stage = T1/T2), with smaller LD by MR (Appendix Figs A1C and A1E).

### Performance of ImPrint in the Training Arm

The pCR rate in unselected patients with HR+HER2– early-stage breast cancer in the first PD-1 inhibitor arm of the trial, pembrolizumab/paclitaxel followed by preoperative AC, was 30%, compared with 13% in concurrent controls.^7^ Stratified by ImPrint, pCR rates were 77% in ImPrint+ versus 7% in ImPrint– (OR, 35; *P* = 1.8E-05; Figs 2B-2D). In this arm, used as the training set for the immune classifier, ImPrint predicted pCR with 83% sensitivity and 89% specificity (PPV = 77%; NPV = 93%).

### Performance of ImPrint in Four Independent Validation Arms

A total of 164 patients with HR+HER2– disease were treated across four subsequent IO arms (durvalumab/olaparib, pembrolizumab/SD-101, cemiplimab, and cemiplimab/LAG3), where ImPrint was evaluated for predictive performance. The overall pCR rate in these four IO arms, taken together, was 34% (56/164) versus 14% (27/191) in the control arm. pCR rates were 74% in ImPrint+ versus 19% in ImPrint– (OR, 12; *P* = 5.1E-11; Figs 2E-2G). In these arms combined, ImPrint predicted pCR with 61% sensitivity and 89% specificity (PPV = 74%; NPV = 81%).

### Performance of ImPrint Varied by Arm

**FIG 3.**
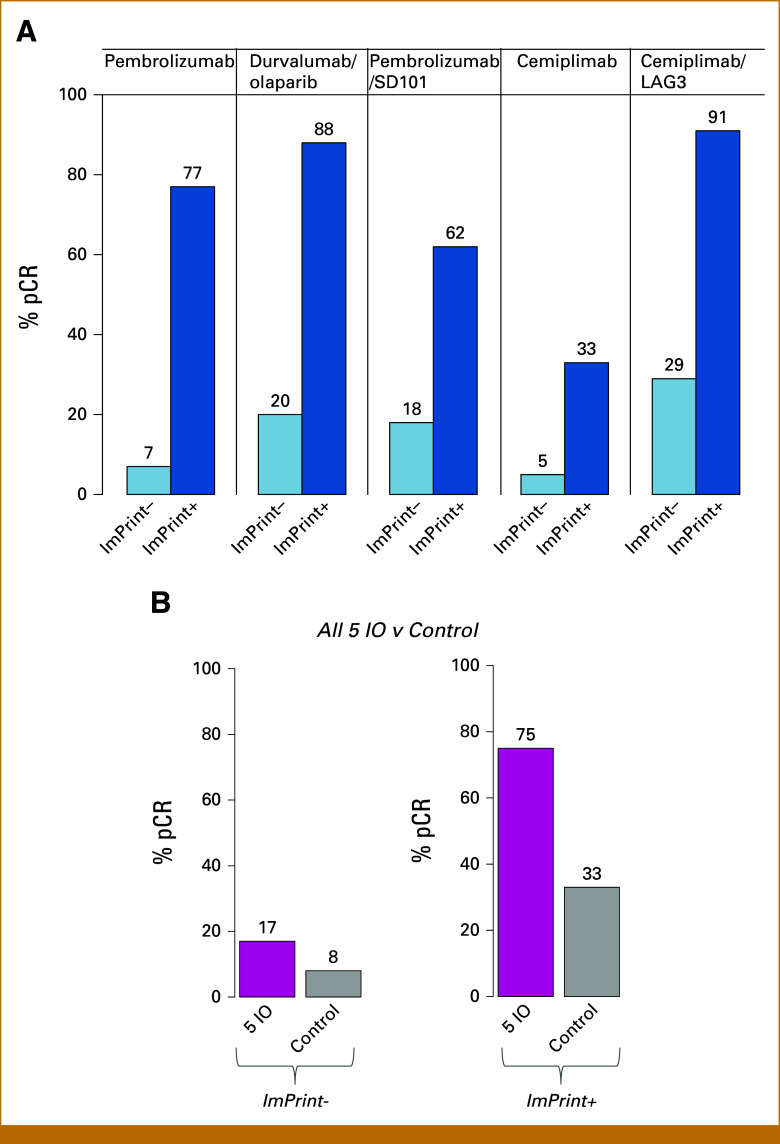
ImPrint performance variations by IO arm and comparison with control. (A) Bar plots showing pCR rates by arm in ImPrint– and ImPrint+ subsets. (B) pCR rate bar plots in all five IO arms together versus control in ImPrint– and ImPrint+ subsets. IO, immunotherapy; LAG3, lymphocyte activation gene 3; pCR, pathologic complete response.

Nominal performance of ImPrint varied by IO arm (Fig 3A), where variance is influenced by small sample sizes (n = 28-51), and dual targeting seems to provide more benefit for some IO treatments. pCR ORs between ImPrint+ and ImPrint– are high (OR, 20 [6.82 to 35]) and significant for most arms (Table 2). The highest pCR rate for HR+HER2–/ImPrint+ exceeded 90% and was observed in a dual-IO arm.

**TABLE 2.** ImPrint Performance in HR+HER2–

Arm/Subset	N	ImPrint– pCR Rate, %	ImPrint+ pCR Rate, %	OR (95% CI)	*P*	Sensitivity, %	Specificity, %	PPV, %	NPV, %
IO training set arm									
Pembrolizumab*	40	7	77	35 (4.8 to 484)	1.8E-05	83	89	77	93
IO validation arms									
All four IO validation arms	164	19	74	12.1 (5.2 to 30.2)	5.1E-11	61	89	74	81
Three IO validation arms that graduated in HR+HER2–	136	22	80	13.92 (5.3 to 40.6)	3.1E-10	60	90	80	78
Durvalumab/Olaparib*	51	20	88	25.6 (4.4 to 282)	7.3E-06	67	93	88	80
Pembrolizumab/SD-101*	46	18	62	6.82 (1.4 to 38)	.01	57	84	62	82
Cemiplimab	28	5	33	9.27 (0.4 to 622)	.11	67	84	33	95
Cemiplimab/LAG3*	39	29	91	22.8 (2.6 to 244)	7.8E-04	56	95	91	71
IO overall									
All five IO arms	204	17	75	14.5 (6.7 to 33)	4.7E-15	65	89	75	83
*All four IO arms that graduated in HR+HER2–	176	19	79	16.2 (7 to 40.7)	3E-14	65	90	79	81
Control arm (T–>AC)									
Control arm	191	8	33	5.5 (2.2 to 14)	9.8E-05	56	82	33	92

Abbreviations: AC, doxorubicin/cyclophosphamide; HR+HER2–, hormone receptor–positive and human epidermal growth factor receptor 2–negative; IO, immunotherapy; LAG3, lymphocyte activation gene 3; NPV, negative predictive value; OR, odds ratio; pCR, pathologic complete response; PPV, positive predictive value.

### Comparison With the Control Arm

14% (27/191) of HR+HER2– patients in the control arm achieved pCR. Even with chemotherapy alone, pCR rates were higher in ImPrint+ (33%) compared with ImPrint– (8%) cancers (Fig 3B; OR, 5.5; *P* = 9.8E-05; Table 2). However, the association is much more dramatic for IO, where the difference in pCR between ImPrint+ and ImPrint– is 55% (75%-17%) versus a difference of 25% (33%-8%) between these subtypes for control treatment (Fig 3B). The biomarker × treatment interaction for ImPrint was significant in the training IO arm versus control (*P* = .045) and showed a trend in five IO arms combined versus control (*P* = .089). Within ImPrint+ tumors, with a pCR rate of 33% in the control arm versus 75% in the combined IO arms, the OR for pCR in IO versus control arms was 5.75 (2.3 to 15.1) (*P* = 5.04E-05; Fig 3B and Table 2). Within ImPrint– tumors, the pCR rate was low in both control and IO arms, though still significantly higher in IO (17%) than in control (8%) (OR, 1.22 [1.01 to 5.1]; *P* = .034).

### Comparison of ImPrint With Tumor Grade, MP1/MP2, ER-Low, and %PD-L1+ as a Predictor of Response to IO

**FIG 4.**
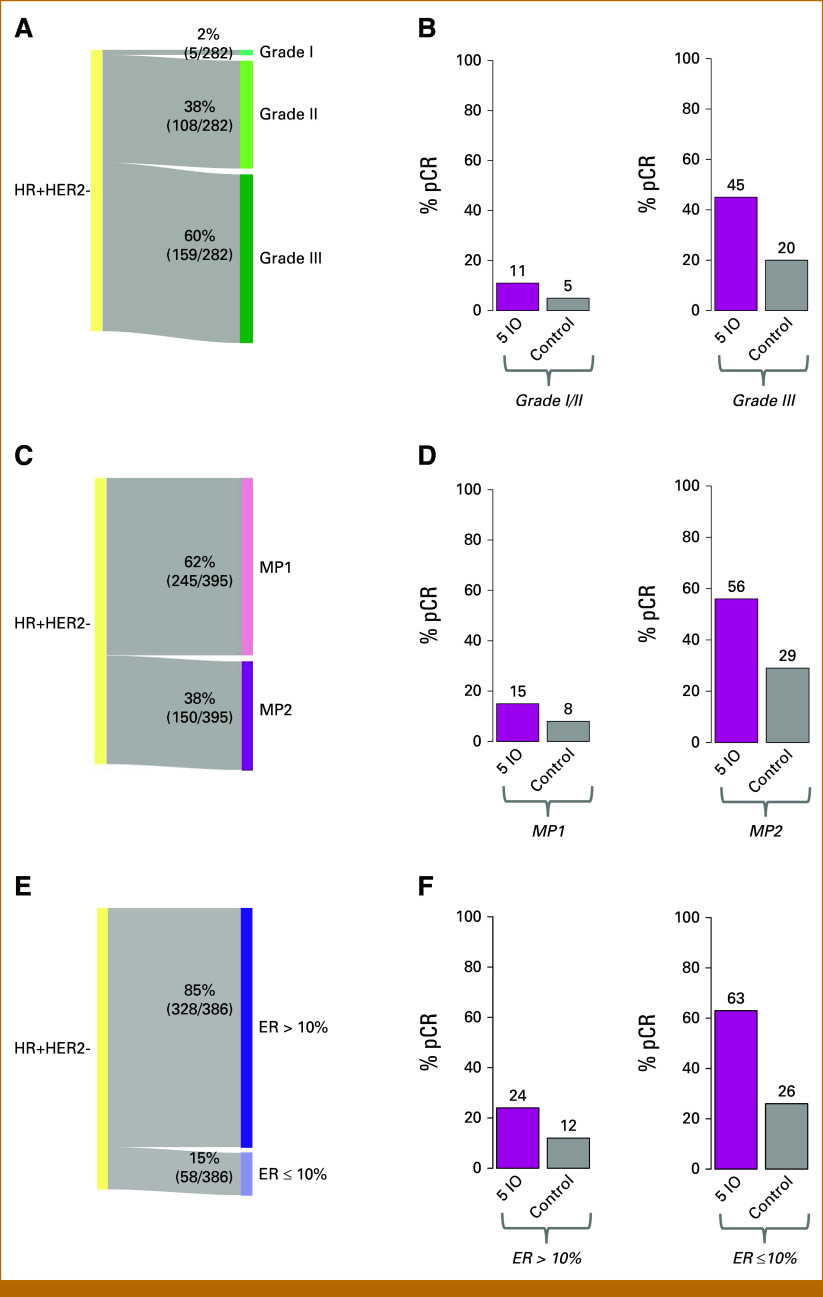
Prevalence and IO response prediction performance of tumor grade, MP1/MP2 and ER-low. (A) Sankey diagram showing prevalence of tumor grade I, II, and II tumors in HR+HER2– in I-SPY2. (B) pCR rate bar plots in all five IO arms together versus control within grade I/II and grade III subsets. (C) Sankey diagram showing prevalence of MP1 versus MP2 class tumors. (D) pCR rate bar plots in all five IO arms together versus control within MP1 and MP2. (E) Sankey diagram showing prevalence of ER-low (ER ≤ 10%) versus other (ER > 10%) tumors. (F) pCR rate bar plots in all five IO arms together versus control within ER-low and ER > 10% tumors. ER, estrogen receptor; HR+HER2–, hormone receptor–positive and human epidermal growth factor receptor 2–negative; IO, immunotherapy; MP1, MammaPrint High 1 risk; MP2, MammaPrint (ultra) High2 risk; pCR, pathologic complete response.

Given that tumor grade (III), MP2 class, ER-low, and PD-L1+ status have been used or proposed for patient selection and efficacy evaluation in recent IO trials in early-stage HR+HER2– breast cancer,^2,3^ we compared their performance with ImPrint. There is overlap between ImPrint+, grade III, MP2, and ER-low status, yet prevalences differ and there are discordances between these markers. Prevalences of ImPrint+, grade III, MP2, and ER-low were 26%, 60%, 38%, and 15%, respectively (Figs 2A, 4A, 4C, and 4E). Discordance between ImPrint+ and MP2 class was 21% (84/395), similar to that of ER-low (20%; 77/386), whereas discordance was higher between ImPrint+ and grade III, at 42% (119/282) (Appendix Fig A1 and Table A1). Our PD-L1 data, currently limited to multiplex immunofluorescence analysis^30^ from patients in the pembrolizumab + paclitaxel arm of I-SPY2,^7^ shows a PD-L1 positivity (>1% PD-L1+ tumor cells) rate of 14% (4/28), and 2 of 4 of the PD-L1+ tumors were ImPrint+.

**FIG A2.**
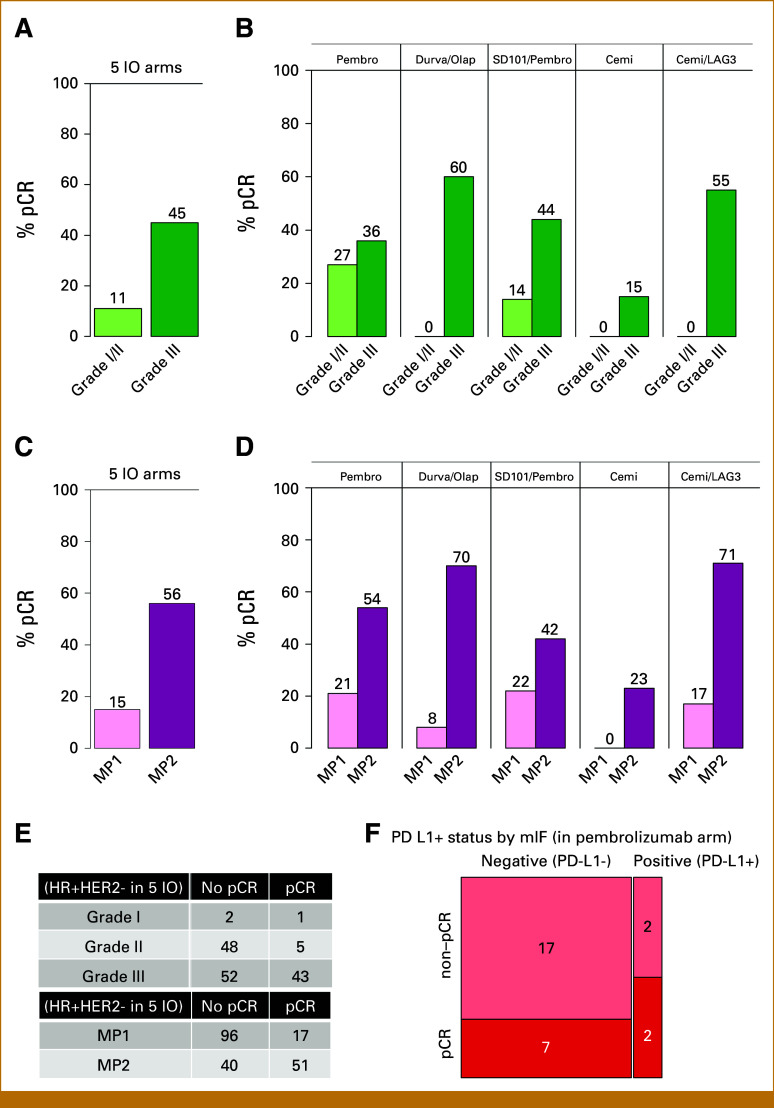
Tumor grade III and MP2 as a predictor of pCR for IO in HR+HER2–. (A and B) Bar plot showing pCR rates in tumor grade III versus grade I/II in the combined five IO arms (A) and within each IO arm (B). (C and D) Bar plot showing pCR rates in MP1/2 class in the combined five IO arms (C) and within each IO arm (D). (E) Tables showing tumor grade versus response (top) and MP1/2 class versus response (bottom) (data for [A] and [D]). (F) Mosaic plot showing pCR as a function of PD-L1-positivity in the pembrolizumab arm by mIF analysis (PD-L1+: %PD-L1+ tumor cells >1%). HR+HER2–, hormone receptor–positive and human epidermal growth factor receptor 2–negative; IO, immunotherapy; LAG3, lymphocyte activation gene 3; mIF, multiplex immunofluorescence; MP1, MammaPrint High 1 risk; MP2, MammaPrint (ultra) High2 risk; pCR, pathologic complete response.

As presented in Table 2 and Figures 2-4, ImPrint+, grade III, MP2, and ER-low all significantly enrich for response to IO (ImPrint: OR, 14.5 [6.7 to 33]; grade III: OR, 6.8 [2.6 to 21.3]; MP High 1 risk [MP1]/MP2: OR, 7.12 [1.36 to 14.8]; ER-low: OR, 5.26 [2.5 to 10]). However, the pCR rates in the combined IO arms for grade III, MP2, and ER-low are 45%, 56%, and 63%, respectively, versus 75% for ImPrint+, with similarly low pCR rates in grade I/II (11%), MP1 (15%), and ImPrint– (17%) cancers, and a 24% pCR rate in ER-low (Fig 4 and Appendix Fig A2 *v* Figs 2 and 3). Although the numbers are very small, the pCR rate for PD-L1+ tumors in the pembrolizumab arm was 50% (2/4) (Appendix Fig A2F).

## DISCUSSION

We previously showed that tumor-immune biology associates with pCR in HR+HER2– patients treated with IO and developed a research-grade immune signature predicting response to IO^17^ that was then translated to a clinically applicable mRNA-based immune classifier (ImPrint) now used in I-SPY2.2 as part of the response predictive subtypes.^25,26^ We extended this work by reporting the performance of ImPrint to predict pCR to neoadjuvant IO therapy in HR+HER2– patients in the IO discovery arm (pembrolizumab) and in four subsequent independent IO validation arms of I-SPY2: durvalumab/olaparib, pembrolizumab/SD101, cemiplimab, and cemiplimab/LAG3, with a comparison with the chemotherapy-only control arm. We also assessed association between ImPrint and clinical/demographic variables and compared its predictive performance with that of other markers that have been used for selection of HR+HER2– patients in other IO trials.

In this high-risk HR+HER2– population, 26% of patients were ImPrint+. There were no statistically significant differences in age, menopausal status, or race/ethnicity between ImPrint± classes. As expected, ImPrint+ tumors had significantly higher grade, ER-low, and MP2 class prevalence than ImPrint– tumors, yet they were significantly smaller, consistent with the hypothesis that immunosurveillance and immunoediting of immune-infiltrated tumors may restrict the size of even highly proliferative cancers.^31^

Within the independent validation IO test set, ImPrint+ cancers had high (74% pCR) and ImPrint– had low (19% pCR) response rates to IO therapy (74% PPV and 81% NPV; OR, 12). These results were similar to those in the population of five IO arms as a whole (ImPrint+: 75% pCR in IO; ImPrint–: 17% pCR in IO).

ImPrint positivity also enriches for response in the chemotherapy-only control arm, albeit much less dramatically than for IO (control: 33% pCR for ImPrint+ *v* 8% pCR for ImPrint–; OR, 5.5). This enrichment for standard chemotherapy response is likely due to shared biological processes, including immune-mediated cytotoxicity, that mediate response to chemotherapy and are enhanced by the addition of an immune checkpoint inhibitor.^32-35^

Given that Imprint positivity predicted a pCR rate of 75% to IO versus 33% to standard-of-care chemotherapy, ImPrint may be a good biomarker to help allocate IO therapy to patients most likely to benefit, as the benefits of IO for ImPrint+ patients seem to greatly outweigh the risks of serious toxicities. By contrast, for patients with HR+HER2– disease who are ImPrint–, there was a much smaller response to IO of 17%, which while greater than the 8% in the chemotherapy-only arm is still quite low. IO agents carry significant risks of immune-related adverse events, some long-lasting (eg, adrenal or thyroid insufficiency), affecting approximately 20%-30% of patients^36-38^ and are costly.^39^ Thus for ImPrint– patients, the (small) benefit of adding IO likely does not outweigh these potential toxicities. By this logic, ImPrint should be clinically useful to guide patients with breast cancer and their providers in making treatment decisions that best balance likely benefit versus potential toxicity, particularly if there are alternative treatments available for ImPrint– predicted IO nonresponders.^17^

Recently, results of two larger randomized neoadjuvant chemotherapy plus IO therapy trials for stage II-III, grade III, ER+HER2– patients were reported. The KEYNOTE-756 trial (ClinicalTrials.gov identifier: NCT03725059) demonstrated improvement in pCR rate with pembrolizumab added to paclitaxel/AC chemotherapy. The pCR rates were 24.3% versus control 15.6% (*P* = .00005) in the overall population, but benefit seemed to be mainly limited to the PD-L1–positive subset of cancers, where pCR rates were 29.7% versus 19.6% in the PD-L1+ and 7.2% versus 2.6% in the PD-L1– subsets.^2^ The CheckMate-7FL trial (ClinicalTrials.gov identifier: NCT04109066), also restricted to patients with grade III tumors, showed an improved pCR rate in the nivolumab plus chemotherapy arm, with 24.5% compared with 13.8% with chemotherapy and placebo (*P* = .0021); again benefit was seen primarily in the predefined PD-L1+ subset, where pCR rates were 44.3% versus 20.2% in the PD-L1+ and 14.2% versus 10.7% in the PD-L1– subsets.^3^ These results are consistent with our findings, although our data are limited to PD-L1+ immunohistochemistry (IHC).

We also compared the performance of ImPrint with tumor grade, which has been used as a selection criterion in IO trials for ER+HER2– patients. ER+HER2– grade III cancers experience higher pCR rates with neoadjuvant chemotherapy with and without IO; however, histologic grade seems to be a suboptimal criterion to select patients for IO therapy (24% pCR rate in KEYNOTE-756 and 45% pCR in I-SPY2). Many grade III cancers are not immune enriched, and these cancers derive little added benefit from IO. ER-low cancers also have higher pCR rates with neoadjuvant chemotherapy^40^ and with IO (63%; this study), but have low prevalence (15%) in even this high-risk cohort with lower response enrichment (OR, 5.3) relative to ImPrint+ (OR, 14.5). PD-L1 positivity with IHC, which correlates with many other immune markers, is a better but still suboptimal patient IO selection tool (ie, pCR rates 30%-44% pCR depending on antibody and assay). Our proposed genomic assay, ImPrint, captures more comprehensive immune activity status to permit more effective selection of IO-sensitive ER+HER2– cancers, as the ImPrint+ cancers have a 75% pCR rate with neoadjuvant IO therapy. MP2, while not quite achieving the performance of ImPrint in I-SPY2, shows a 56% pCR rate with neoadjuvant IO therapy that is superior to that observed using either grade III or PD-L1+. MP2 was previously observed as enriching for pCR in HR+HER2– patients treated with IO and other treatment classes^18,40,41^ and is being prospectively tested in an ongoing randomized trial S2206 (ClinicalTrials.gov identifier: NCT06058377) conducted by the National Cancer Institute Cancer Therapy Evaluation Program and SWOG as a patient selection method for neoadjuvant IO in ER+HER2– cancers.

This study has limitations. ImPrint was developed using expression data from both HR+HER2– and TN patients in the (first) pembrolizumab arm of I-SPY2, and it is possible that using data from HR+HER2– patients exclusively or adding mutational load or neoantigen/diversity might further improve performance. Total IO patient numbers for analysis are reasonable; however, each IO arm is relatively small. This limits the power of analysis. Moreover, different IO arms contain different IO agents with potentially different mechanisms of action (eg, PD-1 *v* PD-L1 inhibitors); some of them in combination with other IO agents (eg, anti-LAG3 or anti–cytotoxic T-cell lymphocyte-4) or other non-IO targeted agents (eg, PARP inhibitor) and were administered with a taxane followed by an anthracycline. For combinations, it is impossible to assess whether a biomarker is predictive of response to an individual agent within the combination or whether it would predict response to IO in the absence of taxane/anthracycline therapy. In I-SPY2.2, one of the innovations is that investigational agents can be tested in a first treatment block without accompanying chemotherapy (I-SPY2.2 trial design, Nat Med commentary, 2024, in press).

Finally, we are limited by a lack of mature distant recurrence-free survival (DRFS) follow-up data for the collected IO arms. It is established that achieving pCR confers excellent prognosis.^12^ However, there is a lack of information regarding the real correlation between non-pCR and outcome in HR+/HER2– patients treated with chemoimmunotherapy. In KEYNOTE-522, addition of pembrolizumab to chemotherapy not only increased pCR rates in TN patients, but also improved event-free survival among TN patients with moderate residual disease (residual cancer burden [RCB]-2).^42^ The extent to which this might be true for HR+HER2– patients and whether it is a result of a downward shift of RCB during neoadjuvant IO or the result of adjuvant IO remains to be seen.

Despite these limitations, this study establishes that a subset of clinically and genomically high-risk, ER+HER2– breast cancers are highly sensitive to IO and using a specific and sensitive selection strategy, patients could achieve pCR rates similar to those achieved with the most effective neoadjuvant chemotherapies in TNBC and HER2+ cancers (ie, pCR rate >65%-70%). ImPrint, an FDA IDE-enabled experimental assay currently being further prospectively evaluated in I-SPY2, may represent the best approach to identify patients for IO to optimally balance likely benefit versus risk of immune-related severe adverse events.

## Data Availability

A data sharing statement provided by the authors is available with this article at DOI https://doi.org/10.1200/PO-24-00776. Clinical and biomarker data from this study are available in Data Supplement.

## APPENDIX 1. I-SPY2 INVESTIGATORS

Rita Mukhtar, MD (University of California San Francisco, San Francisco, CA); Michelle Melisko, MD (University of California San Francisco, San Francisco, CA); Anne Wallace, MD (University of California San Diego Moores Cancer Center, La Jolla, CA); Kay Yeung, MD (University of California San Diego Moores Cancer Center, La Jolla, CA); Kathy Albain, MD (Loyola University Chicago Stritch School of Medicine, Maywood, IL); Patricia Robinson, MD (Loyola University Chicago Stritch School of Medicine, Maywood, IL); Shelley Lo, MD (Loyola University Chicago Stritch School of Medicine, Maywood, IL); Funmi Olopade, MD (University of Chicago Medical Center, Chicago, IL); David Potter, MD (Masonic Cancer Center, University of Minnesota, Minneapolis, MN); Heather Beckwith, MD (Masonic Cancer Center, University of Minnesota, Minneapolis, MN); Anne Blaes, MD (Masonic Cancer Center, University of Minnesota, Minneapolis, MN); Judy Boughey, MD (Mayo Clinic Breast Cancer Center, Rochester, MN); Tufia Haddad, MD (Mayo Clinic Breast Cancer Center, Rochester, MN); Anthony Elias, MD (University of Colorado Cancer Center, Aurora, CO); Claudine Isaacs, MD (Georgetown University, Washington, DC); Zahi Mitri, MD (Health and Science University, Portland, OR); Kathleen Kemmer, MD (Health and Science University, Portland, OR); Janice Lu, MD (University of Southern California, Los Angeles, CA); Julie Lang, MD (University of Southern California, Los Angeles, CA); Alexandra Thomas, MD, FACP (Wake Forest University, Winston-Salem, NC); Meghna Trivedi, MD (Columbia University, New York, NY); Dawn Hershman, MD (Columbia University, New York, NY); Jane Meisel, MD (Emory University Winship Cancer Institute, Atlanta, GA); Kevin Kalinsky, MD (Emory University Winship Cancer Institute, Atlanta, GA); Christos Vaklavas, MD (University of Utah Huntsman Cancer Institute, Salt Lake City, UT); Nicole Williams, MD (Ohio State University Medical Center, Columbus, OH); Erin Ellis, MD (Swedish Cancer Institute, Seattle, WA); Amy Sanford, MD (Sanford Health, Sioux Falls, SD); Tara Sanft, MD (Yale University, New Haven, CT); Rebecca Viscusi, MD (Arizona Cancer Center, Tucson, AZ); Mili Arora, MD (University of California—Davis Comprehensive Cancer Center, Sacramento, CA); Carla Falkson, MD (University of Rochester, Rochester, NY); Donald Northfelt, MD (Mayo Clinic—Scottsdale, Scottsdale, AZ); Rashmi Murthy, MD (MD Anderson Cancer Center, University of Texas, Houston, TX); Barbara Haley, MD (Southwestern Medical Center, University of Texas, Dallas, TX); Rachel Yung, MD (University of Washington, East Seattle, WA); Ingrid Mayer, MD (Vanderbilt University Medical Center, Nashville, TN); Qamar Khan, MD (University of Kansas Medical Center, Westwood, KS); and Kirsten K. Edmiston, MD (Fairfax Hospital Cancer Center, Fairfax, VA)

**TABLE A1.** Demographic and Tumor Characteristics Versus ImPrint Status in HR+HER2–

Clinical/Demographic Variable	Overall (n = 395), N (%) Unless Otherwise Indicated	ImPrint– (n = 291), N (%) Unless Otherwise Indicated	ImPrint+ (n = 104), N (%) Unless Otherwise Indicated	*P* (ImPrint+ *v* ImPrint–; Fisher Exact Test or *t* Test)
Age, years				
Median (range)	47 (19-71)	48 (19-71)	45 (24-71)	.16
Race/ethnicity				
White (non-Hispanic)	270 (68)	203 (70)	67 (64)	.65
White (Hispanic)	51 (13)	35 (12)	16 (15)	
Black	43 (11)	32 (11)	11 (11)	
Asian	25 (6)	17 (6)	8 (8)	
American Indian or Alaska Native	2 (0.5)	1 (0.3)	1 (1)	
Multiple races	2 (0.5)	2 (0.7)	0 (0)	
Unknown	2 (0.5)	1 (0.3)	1 (1)	
Menopausal status				
Premenop/perimenopausal (or age <50)	238 (60)	170 (58)	68 (65)	.24
Postmenopausal (or age >50)	157 (40)	121 (42)	36 (35)	
MP status				
MP1	245 (62)	226 (78)	19 (18)	<**2E-16**
MP2	150 (38)	65 (22)	85 (82)	
Tumor grade (available for n = 282/395)				
Grade I	5 (2)	5 (2)	0	**4.3E-07** (*P* for unknown *v* known grade is .13)
Grade II	108 (38)	99 (46)	9 (13)	
Grade III	169 (60)	110 (51)	59 (87)	
Unknown	113	77	36	
Tumor longest diameter on MR, cm				
Median (range)	3.9 (1.3-13)	4 (1.3-13)	3.7 (1.4-11.4)	**.0039**
% ER+ cells (available for n = 386/395)				
ER ≤ 10%	58 (15)	19 (7)	39 (40)	<**2E-16**
ER > 10%	328 (85)	270 (93)	58 (60)	
Unknown	9	2	7	
Tumor stage (T)				
T1	12 (3)	6 (2)	6 (6)	**.0082**
T2	238 (62)	165 (58)	73 (71)	
T3	126 (33)	104 (37)	22 (21)	
T4	10 (2.6)	8 (3)	2 (2)	
Unknown	9	8	1	
Nodal stage (N)				
N0	135 (35)	93 (33)	42 (41)	.26
N1	204 (53)	157 (56)	47 (46)	
N2	20 (5)	15 (5)	5 (5)	
N3	25 (7)	16 (6)	9 (9)	
Unknown	11	10	1	

Abbreviations: ER, estrogen receptor; HR+HER2–, hormone receptor–positive and human epidermal growth factor receptor 2–negative; MP1, MammaPrint High 1 risk; MP2, MammaPrint (ultra) High2 risk; MR, magnetic resonance (image).
